# *Banha-sasim-tang *as an herbal formula for the treatment of functional dyspepsia: a randomized, double-blind, placebo-controlled, two-center trial

**DOI:** 10.1186/1745-6215-11-83

**Published:** 2010-07-30

**Authors:** Jae-Woo Park, Bongha Ryu, Inkwon Yeo, Ui-Min Jerng, Gajin Han, Sunghwan Oh, Jinsoo Lee, Jinsung Kim

**Affiliations:** 1 Department of Gastroenterology, College of Oriental Medicine, Kyung Hee University, Seoul, Korea; 2 Department of Statistics, College of Sookmyung Women's University, Seoul, Korea

## Abstract

**Background:**

Functional dyspepsia (FD) is characterized by a high prevalence rate and no standard conventional treatments. Alternative therapies, such as herbal formulas, are widely used to treat FD. However, there are inadequate evidences regarding the safety and efficacy of these formulas. Moreover, the mechanisms by which herbal formulas act in the gastrointestinal tract are controversial. In traditional Korean medicine, *Banha-sasim-tang *has long been one of the most frequently prescribed herbal formulas for treating dyspepsia. The current study is designed to evaluate the efficacy and safety of *Banha-sasim-tang *for FD patients and to examine whether there will be a significant correlation between cutaneous electrogastrography recordings and dyspeptic symptoms in FD patients, and between changes in gastric myoelectrical activity and improvement in dyspeptic symptoms during *Banha-sasim-tang *administration.

**Methods:**

This randomized, double-blind, placebo-controlled trial will be performed at two centers and will include a *Banha-sasim-tang *group and placebo group. Each group will consist of 50 FD patients. Six weeks of administration of *Banha-sasim-tang *or placebo will be conducted. During the subsequent 2 months, follow-up observations of primary and secondary outcomes will be performed. The primary outcomes are differences as measured on the gastrointestinal symptom scale, and the secondary outcomes are differences as measured on the visual analogue scale for dyspepsia and on the questionnaire for FD-related quality of life. All outcomes will be measured at baseline, at 2, 4, and 6 weeks of treatment, and at the 1 and 2 month follow-up. Cutaneous electrogastrography will be performed and assessed at baseline and at 6 weeks.

**Discussion:**

This trial will provide evidence of the safety and efficacy of *Banha-sasim-tang *for the treatment for FD. Furthermore, based on the assessment of the relationship between cutaneous electrogastrography recordings and dyspeptic symptoms in this trial, the possibility of clinical applications of cutaneous electrogastrography in the treatment of FD will be elucidated.

**Trial Registration:**

Current Controlled Trials (ISRCTN 51910678); Clinical Trials.gov Identifier: NCT00987805

## Background

Functional dyspepsia (FD) is characterized by chronic or relapsing dyspeptic symptoms in the absence of structural lesions that can be identified with clinically available tests [1,2]. In developed countries, 15-20% of the general population experiences dyspeptic symptoms at some point over the course of any given year [3]. An epidemiologic survey conducted in South Korea reported that 25% of the population suffers from FD [4].

Although the pathogenic causes of FD remain unclear, delayed gastric emptying has been found in up to 50% of FD patients [5]. Delayed gastric emptying may be attributed to gastric hypomotility and to uncoordinated antral duodenal contractions [6]. Normal gastric slow waves originating in the gastric pacemaker lead to normal frequency and peristaltic gastric contractions [5]. Abnormal gastric myoelectrical dysrhythmias has been observed in FD patients who have shown delayed gastric emptying [5]. Cutaneous electrogastrography (EGG) is a non-invasive diagnostic technique that detects gastric myoelectrical activity (GMA). Many researchers have used cutaneous EGG, which suggests that this technique may be useful in the evaluation of gastric motor function in FD patients [7]. However, the relationship between dyspeptic symptoms and cutaneous EGG recordings remains a controversial topic in FD.

Current treatments for FD target putative underlying mechanisms, including visceral hypersensitivity, impaired gastric emptying, and acid hypersensitivity [8]. The symptoms of FD are diverse, thus mechanism-focused therapies, such as acid secretion inhibitors, prokinetics, and *H. pylori *eradication, have been used with limited effects [8-10]. Therefore, many patients use alternative therapies, including herbal formulas, acupuncture treatments, and natural products, to treat FD [2,11-16].

*Banha-sasim-tang *(BST; *Hange-shashin-to *in Kampo Medicine; *Banxia-xiexin-tang *in Traditional Chinese Medicine) is one of the herbal formulas described in "Treatise on Cold Damage and Miscellaneous Diseases (*Shan-han-za-bing-lin*)" [17], the Chinese authoritative monographs. This formula is composed of seven herbs. In traditional Korean medicine, this formula has been applied for treating the symptom "gastric stuffiness" [17], which is similar to dyspepsia. Recently, several studies have elucidated the gastric function and related mechanisms of BST [18-20].

Moreover, BST can be obtained as an over-the-counter herbal formula in Korea or prescribed for dyspeptic symptoms by the Traditional Korean Medicine doctors. For that reason, reliable clinical evidence regarding BST as treatment for FD is needed. However, there are no relevant randomized controlled clinical trials regarding FD as far as we know.

The current study is designed to investigate the effect of BST on FD and related quality of life. We will also examine the relationship between the frequency or power variables in cutaneous EGG and dyspeptic symptoms of FD patients in this trial and determine whether the changes in GMA recorded by cutaneous EGG before and after the oral administration of BST can reflect the clinical efficacy of BST in the treatment of FD.

## Methods

### Objectives

The aims of this study are to:

(1) To determine whether BST can improve dyspeptic symptoms in patients with FD.

(2) To examine the relationship between dyspeptic symptoms and cutaneous EGG recordings and a possible biological evidence of BST's efficacy via cutaneous EGG recordings.

### Hypothesis

(1) Six weeks of oral administration of BST improves dyspeptic symptoms and quality of life in patients with FD.

(2) In patients with FD or FD subtypes according to the Rome III criteria, there will be a significant correlation between the degree of dyspeptic symptoms and cutaneous EGG recordings and 6 weeks of oral administration of BST can improve the abnormal frequency and power parameters on cutaneous EGG.

### Design

This study will be carried out as a randomized, placebo-controlled, double-blind, two-center trial at the Oriental Hospital at Kyung Hee University Medical Center and at the Oriental Hospital at the East-West Neo Medical Centre of Kyung Hee University in Seoul, Korea.

**Figure 1:**
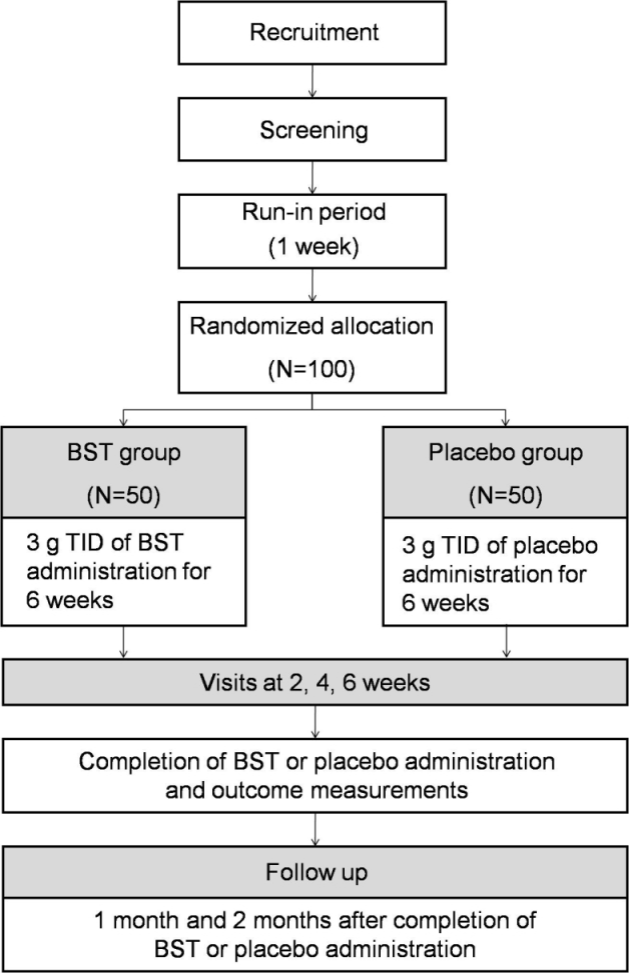
**Flow chart of the trial**.

This clinical trial will consist of a 6-week oral administration of BST and a 2-month follow-up period. Before screening, all participants will go through a 7-day washout phase. During 6 weeks' administration of experimental drugs, patients will be prohibited from taking any kind of dyspepsia-relieving drugs. After randomization, 3 g TID of BST or placebos will be provided for 6 weeks. Outcomes will be measured at baseline, at 2 weeks, at 4 weeks and at 6 weeks after randomization. Outcomes will also be measured at 1 month and 2 months after completion of BST administration. During 2-month follow-up period, conventional treatments for dyspepsia will be permitted if the dyspeptic symptoms are exacerbated or recur. Any treatment received by the patient during the follow-up period will be reported by them or documented in their diary (Figure 1).

This study will be performed in accordance with the standards of the International Committee on Harmonization on Good Clinical Practice and the revised version of the Declaration of Helsinki. The protocol of the trial has been approved by two ethics committees: the institutional review boards of both the Oriental Hospital at Kyung Hee University Medical Center and the Oriental Hospital at East West Neo Medical Center of Kyung Hee University. The permission numbers are KOMC IRB 2009-05 for the Oriental Hospital at Kyung Hee University Medical Centre and KHNMC-OH-IRB 2009-001 for the Oriental Hospital at East-West Neo Medical Centre of Kyung Hee University. Written informed consent will be obtained from all participants prior to enrollment, and patients will be given enough time to decide if they wish to participate before signing the consent form.

### Participants

#### Inclusion criteria

Patients 19-75 years old who complain of dyspepsia for the previous 3 months, and who have an onset of symptoms at least 6 months prior, meet the definition of the Rome III criteria for FD [21]. Patients with one or more of the following symptoms: postprandial fullness, early satiety, epigastric pain or burning, will be also considered as meeting the study definition of FD. Then, all the participants will undergo endogastroduodenoscopy (EGD) before enrollment and be examined by gastroenterologists to determine whether the EGD observations are related to the present dyspeptic symptoms. On the other hand, the *Helicobacter pylori *status of the patients and history of *H. pylori *eradication therapy will be assessed before enrollment by non-invasive tests (urea breath test) or the rapid urease test.

Patients who are diagnosed with FD can be categorized as having either 1) meal-induced dyspeptic symptoms (postprandial distress syndrome; PDS) or 2) epigastric pain syndrome (EPS) and, in this trial, all FD patients will be classified into one of the abovementioned subtypes (PDS and EPS) [21]. The dyspepsia severity of patients who meet the Rome III criteria will be assessed by a validated Gastrointestinal Symptom (GIS) scale, which measures the severity of 10 symptoms [22]. The presence of "moderate" as the degree of severity for at least three GIS scale symptoms will be a basic criterion for trial inclusion [2].

#### Exclusion criteria

Patients who report the following conditions will be excluded:

History of peptic ulcer, gastroesophageal reflux disease (GERD), gastrointestinal surgery or mental disorders, marked current symptoms of irritable bowel disease, the presence of the alarm symptoms such as severe weight loss, black or tar stool, dysphagia, the presence of uncontrolled severe organ diseases including cancer, ingestion of aspirin or nonsteroidal anti-inflammatory drugs (NSAIDs), and women who are pregnant or lactating.

At the screening phase, patients who are using any antibiotic, proton-pump inhibitor, bismuth salt, prokinetic agents such as itopride, herbal formulas, or who are participating in any other clinical trial, will be excluded from this study.

### Recruitment

Recruitment will be done through hospitals and with newspaper advertisements. Patients who have severe dyspeptic symptoms are likely to receive conservative therapies. Thus, interested patients with severe symptoms of functional dyspepsia may be recruited through hospitals. However, patients with mild to moderate symptoms of functional dyspepsia seldom receive regular hospital treatment. Recruitment advertisements in newspapers can attract those patients with less severe symptoms.

### Randomization

Randomization will be controlled by an independent clinical research coordinator (CRC). First, the randomization form with the basic information of the participant who has passed the screening phase will be transmitted in facsimile to the independent statistician. The randomization number in the randomization form will be left blank before arrival to the statistician. The statistician will then decide the randomization number based on the allocation sequence which has been generated by a random number creation program in advance and the statistician will return the randomization form filled in with the established number (specific ID number) to the CRC. The ratio of randomization allocation to the sites will be 1:1. The CRC will inform the investigators of the specific ID number. This procedure will be guaranteed by the authorized contract research organization (CRO).

### Blinding

In this trial, investigators will not be in contact with the CRC, the clinical pharmacist, or the statistician. The CRC will be separated from all researchers, thus the researchers will not have any influence on enrollment or randomization. The statistician will receive the randomization form in facsimile, fill in the blank and return it in order, thus any contact with other researchers cannot be made. The blinding procedure will also be verified by the authorized CRO.

### Experimental drugs

#### Banha-sasim-tang (BST)

BST has long been used in traditional Korean medicine to treat gastrointestinal diseases. According to recent experimental researches, BST has been known to reduce inflammation in inflammatory bowel diseases and diarrhea, to regulate gastrointestinal functional in FD, and to protect against the side effects of chemotherapy in gastrointestinal cancers [18-20,23,24].

Although many experimental studies suggest that BST can be used to treat FD, there are no randomized studies of BST as a treatment for FD as far as we know.

BST used in this trial is a brown, bitter herbal extract granule (Bansasin granule^®^, Hanpoong Pharm & Food Co., Ltd., Jeonju, Korea) produced according to Korean Good Manufacturing Practice. Bansasin granule^® ^is permitted and regulated by the Korean Food & Drug Administration. Each 3 g Bansasin granule^® ^(water-extracted BST mixed with starch and lactose) is composed of seven herbs: *Pinelliae Tuber *(the rhizome of *Pinellia ternata *(Thunb.) Breit., family Araceae) 1.67 g, *Scutellariae Radix *(the root of *Scutellaria baicalensis *Georgi, family Labiatae) 1.00 g, *Ginseng Radix *(the root of *Panax ginseng *C.A. Meyer, family Araliaceae) 1.00 g, *Glycyrrhizae Radix *(the root of *Glycyrrhiza uralensis *Fisch., family Leguminosae) 1.00 g, *Zizyphi Fructus *(the fruit of *Zizyphus jujuba *Mill. var. inermis Rehder, family Rhamnaceae) 1.00 g, *Zingiberis Rhizoma *(the rhizome of *Zingiberis officinale *Roscoe, family Zingiberaceae) 0.83 g, and *Coptidis Rhizoma *(the rhizome of *Coptis chinensis *Franch., family Ranunculceae) 0.33 g. As standard chemical components in each 3 g Bansasin granule^®^, 11.6 mg of berberin, 25.0 mg of glycyrrhzin acid and 100.0 mg of baicalin are included. Voucher specimens will be retained at the research laboratory of Hanpoong Pharm & Food Company. Regular dosage is 3 g TID for adults while dyspeptic symptoms, nausea, vomiting, diarrhea, abdominal pain, and anorexia continue.

#### Placebo

At present, there is no standard treatment in FD. Thus, the placebo for this trial requires no active components. Although Bansasin granule^® ^is not a chemical drug and relevant drug packagings may be needed for succeeding blinding, drug packaging, such as starch capsules, will not be used for reproducing the real situation of Bansasin granule^® ^administration in this trial. Therefore, mimicking the original color and taste of the Bansasin granule^® ^without adding any active components was quite a difficult procedure. The company that makes the Bansasin granule^® ^was experienced in making placebos of herbal extracts and they succeeded in making a Bansasin granule^® ^placebo through the development of several test samples. The placebo is a starch and lactose mixture which has a color and taste similar to the Bansasin granule^®^. Retention samples of placebo used in the current study will be kept at the Hanpoong Pharm & Food Company. At the end of the study, the question to participants whether the drugs that they have taken is real or not will be answered by themselves for evaluation of success in blinding.

### Outcome measures

#### Primary outcome

The primary outcome is the proof of BST's superiority compared with placebo in treating FD. For this purpose, the GIS scale is chosen as the primary variable [1]. The primary efficacy parameter is the change in the sum totals of the GIS scales. The GIS scale is composed of the following 10 dyspeptic symptoms: epigastric pain/upper abdominal pain, abdominal cramps, fullness, early satiety, loss of appetite, malaise, nausea, vomiting, retrosternal discomfort, and acidic regurgitation/heartburn. Symptom severity per each item will be assessed by a 5-point Likert scale: none - 0, slight - 1, moderate - 2, severe - 3, and very severe - 4. The GIS scale is very easy for participants to understand and to complete. The GIS scale will be assessed at baseline, 2 weeks, 4 weeks and 6 weeks during oral administration of BST, and at 1 month and 2 months after completion of BST administration.

#### Secondary outcomes

A visual analogue scale (VAS) will be used to determine the patient's global judgment of intensity of discomfort due to dyspepsia (ranging from 0 mm as no discomfort to 100 mm as the most intense discomfort). The VAS measurements will be performed with the same frequency as the GIS scale measurements.

The validated Functional Dyspepsia related Quality of Life (FD-QoL) questionnaire assesses FD's influence on quality of life and consists of four categories: diet (5 items), daily activity (4 items), emotion (6 items), and social functioning (6 items) [25]. FD-QoL will also be performed with the same frequency as the GIS scale measurements.

### Measurement of GMA

Gastric hypomotility and uncoordinated antral duodenal contractions in FD patients are closely associated with gastric myoelectrical dysrhythmias. These dysrhythmias arise from dysregulation of gastric slow waves, which normally occur at a frequency of 3 cycles per minute (cpm) [26].

Electrogastrogram records GMA acquired from cutaneous abdominal electrodes [27]. Although some researchers have suggested that some cutaneously acquired dysrhythmias may be artifactual in nature [28], other experiments have suggested a positive correlation between the frequencies found with cutaneous EGG recordings and myoelectrical signals acquired from gastric serosal leads [29,30].

In this study, the GMAs of the participants will be measured using surface multichannel EGG (Polygraf ID^®^, Medtronic A/S, Denmark) at baseline and at 6 weeks. This method of EGG measurement will be conducted as described previously [26]. First, the epigastric skin to be attached to the electrodes will be shaved and abraded with a sandy skin preparation jelly to reduce impedence. Four active surface electrodes will be positioned at the sites: the corpus of stomach as channel 1, proximal antrum as channel 2, distal antrum as channel 3, and pylorus region as channel 4. A ground electrode and a reference electrode will also be placed. EGG measurements will be performed in a quiet room and patients will fast over night for ≥ 8 hours. Participants will be asked not to talk and to remain as still as possible during the EGG assessment to avoid motion artifacts. Patients will undergo a 20 minute fasting (preprandial) EGG measurement in the supine position, then they will eat two scrambled medium eggs and two pieces of toasted bread with 500 ml of water as the standard solid test meal (500 Kcal). Then, postprandial EGG measurement will be conducted for 40 minutes. The percentage of slow wave coupling, the EGG dominant frequency and power, the percentage of normal gastric slow waves, the percentage of gastric dysrhythmia, and the postprandial to preprandial power ratio will be assessed.

### Safety

Before randomization and after completion of BST administration, we will perform the following tests on all participants: complete blood cell count, AST/ALT, γGT, BUN, creatinine, erythrocyte sedimentation rate as well as electrocardiogram. The above tests will serve to exclude participants who have serious illnesses and abnormal heart, liver, kidney, or other organ functioning. Throughout the study, we will also assess whether 6 weeks of BST administration in FD patients is safe by above tests or CRF documentations.

The guide for taking BST which are verified by the Korean Food & Drug Administration lists several adverse events: pseudoaldosteronism, myopathy, skin disorders, liver malfunction, pneumonia, or dry mouth. During the trial, all adverse events will be observed in detail and documented in case report forms (CRFs).

### Quality control

Before starting the trial, investigators who assess the EGG will receive thorough training in taking EGG measurements.

To maintain the accuracy and quality of the clinical trial, audit and monitoring will be conducted by the Marinet Corporation, a CRO located in Seoul, Korea. The sites' CRF completion and compliance with standard operation procedures will be audited. Clinical research associates will, at regular periods, monitor the clinical trial procedures such as compliance with BST administration and voluntary withdrawal of participants. In particular, reasons for withdrawal will be fully documented in CRFs.

### Statistical analysis

The primary hypothesis is that oral administration of BST is more effective than placebo for treating FD. We hope to prove this hypothesis by means of a 2-sided test yielding a 5% significance level. Because there is no relevant previous study using BST for calculating sample size, we referred to a similar herbal trial for FD treatment which used the GIS scale [1]. The formula for estimating the sample size is as follows:

The previous trial demonstrated 3.5 points of improvement (*μ*_*c *_- *μ*_*t *_= *Δ*) in the GIS scale over treatment with placebo during 4 weeks of herbal treatments [1]. The same study indicated a mean standard deviation (SD = *σ*) of 5.37. In our study, the ratio (*λ*) of experimental group to placebo group will be 1:1. With a power of 80% (1 - β) and significance level of 5% (α), assuming Δ = 3.5 and σ = 5.37, a sample size of *n_t _*= *n_c _*= 37 patients per treatment group will be required. (*n_t_*, number of BST group; *n_c_*, number of placebo group). Considering an assumed dropout rate of 25%, a total of 100 patients will be needed.

The analysis strategy in this study is as follows:

As a first step, the baseline characteristics of both groups, sex, age, duration of dyspeptic periods, and smoking, will be compared. As a second step, we will compare the efficacy of BST and placebo, as the change in GIS totals from the beginning (0 day) to the end (6 weeks) of the study period. As a third step, we will analyze the secondary variables (VAS and FD-QoL) in the same manner as we did the GIS scale. Finally, various parameters in the EGG measurement, such as frequency and power-related variables will be compared before and after treatment in both groups. Correlations between changes in GIS scale results and EGG parameter findings will also be analyzed.

All analyses in this study will be based on the intention-to-treat principle. If data distribution is skewed owing to insufficient sample size, relevant transformation by a statistician prior to analysis will be made. The baseline characteristics will be compared by either *χ*^2^-test or the Student *t*-test. Primary and secondary outcomes will be presented as means and SDs, and analyzed by independent *t*-tests, Mann-Whitney tests, or Wilcoxon singed-rank tests. Correlations between the GIS scale and EGG parameters will be analyzed by Pearson's correlation coefficients or by Spearman's Rho. Adverse events will be calculated and compared using *χ*^2^-test or Fisher's exact test.

Statistical analyses will be conducted in a blind manner by an independent statistician and performed using SPSS 16.0 (SPSS inc., Chicago, Illinois, USA).

## Discussion

The current clinical study is a randomized, double-blind, placebo-controlled trial investigating the safety and efficacy of frequently used herbal formulas as part of the national project for studying the traditional herbal medicines of South Korea. This clinical trial belongs to the 2009 Traditional Korean Medicine R&D projects funded by the Ministry for Health & Welfare & Family Affairs, Republic of Korea. BST is one of the most frequently prescribed herbal formulas in Korea, BST is thought to be safe, and experimental evidences support BST's effectiveness for dyspepsia [18-20]. However, there have not been any randomized controlled trials showing the effectiveness of BST for the treatment of FD. Through the current study, we expect to gain objective clinical evidences of the efficacy and safety of BST for the treatment of FD.

The causes of functional dyspepsia are diverse and overlapping. Of the putative causes of FD, delayed gastric emptying/gastric dysmotility is one of the most important causes of FD in Korea and has been reported to closely correlate to GMA recorded by cutaneous EGG [5]. Because many researchers have suggested that cutaneous EGG could be a relatively easy technique that can be used to assess GMA in clinical settings and EGG recordings showed a significant correlation with dyspeptic symptoms in FD patients and changes in cutaneous EGG parameters in FD patients could reflect changes in their gastric motility and their dyspeptic symptoms [29,30], it is postulated that a significant correlation can be found between dyspeptic symptoms of FD patients and cutaneous EGG recordings in this trial, and the improvement in dyspeptic symptoms after the oral administration of BST may be assessed by measuring the changes in cutaneous EGG parameters. Correlations in these parameters can evaluate the possibility of clinical applications of cutaneous EGG in the treatment of FD, and a detailed guide, based on biological evidences, for treatment in FD patients.

## Abbreviations

FD: functional dyspepsia; EGG: electrogastrography; EGD: endogastroduodenoscopy; *H. pylori*: *Helicobacter pylori*; PDS: postprandial distress syndrome; EPS: epigastric pain syndrome; GMA: gastric myoelectrical activity; BST: *Banha-sasim-tang*; GIS: gastrointestinal symptom; GERD: gastroesophageal reflux disease; NSAIDs: nonsteroidal anti-inflammatory drugs; CRC: clinical research coordinator; CRO: contract research organization; VAS: visual analogue scale; FD-QoL: functional dyspepsia related quality of life; CRF: case report form.

## Competing interests

The authors declare that they have no competing interests.

## Authors' contributions

JSK, JWP, and BHR contributed to the securing of funding for the project and to the study design. UMJ, GJH, SHO and JSL participated in the design of the trial. JSK and JWP drafted the protocol and wrote the final manuscript. JWP and IKY were responsible for the statistical design of the trial. All authors read and approved the final manuscript.
